# Idiosyncratic media exposures during a pandemic and their link to well-being, cognition, and behavior over time

**DOI:** 10.1073/pnas.2304550120

**Published:** 2023-06-20

**Authors:** Nickolas M. Jones, Rebecca R. Thompson, E. Alison Holman, Roxane Cohen Silver

**Affiliations:** ^a^Department of Psychological Science, University of California, Irvine, CA 92697; ^b^Sue & Bill Gross School of Nursing, University of California, Irvine, CA 92697; ^c^Department of Medicine, University of California, Irvine, CA 92697; ^d^Program in Public Health, University of California, Irvine, CA 92697

**Keywords:** COVID-19, information channels, distress, risk behavior, news media

## Abstract

Over the past two decades of research, increased media consumption in the context of collective traumas has been cross-sectionally and longitudinally linked to negative psychological outcomes. However, little is known about the specific information channels that may drive these patterns of response. The current longitudinal investigation uses a probability-based sample of 5,661 Americans measured at the onset of the COVID-19 pandemic to identify a) distinct patterns of information-channel use (i.e., dimensions) for COVID-related information, b) demographic correlates of these patterns, and c) prospective associations of these information channel dimensions with distress (i.e., worry, global distress, and emotional exhaustion), cognition (e.g., beliefs about the seriousness of COVID-19, response efficacy, and dismissive attitudes), and behavior (e.g., engaging in health-protective behaviors and risk-taking behaviors) 6 mo later. Four distinct information-channel dimensions emerged: journalistic complexity; ideologically focused news; domestically focused news; and nonnews. Results indicate that journalistic complexity was prospectively associated with more emotional exhaustion, belief in the seriousness of the coronavirus, response efficacy, engaging in health-protective behaviors, and less dismissiveness of the pandemic. A reliance on conservative-leaning media was prospectively associated with less psychological distress, taking the pandemic less seriously, and engaging in more risk-taking behaviors. We discuss the implications of this work for the public, policy makers, and future research.

## Significance

During the early onset of the COVID-19 pandemic, information about the novel coronaviruses’ threat permeated the news-media sphere. Over time, however, messaging and analysis of the threat began to fracture across the media landscape as pandemic mitigation efforts became politicized and experts weighed in on the emerging data on transmission and risk. We show how the media channels people relied on for COVID-related information at the onset of the pandemic are linked with distress, belief in the seriousness of the threat, and efficacy of mitigation efforts, as well as health-protective and risk-taking behaviors 6 mo later. Our results reveal that news consumers should broaden their media diets to included reputable news sources to form a better understanding of novel threats.

The news media serve a critical function in disseminating information to the public when large-scale traumatic events unfold. Such events are often ambiguous (1–3) and the uncertainty people feel when they strike initiates information-seeking behavior in an effort to mitigate psychological discomfort (4); this behavior continues particularly when the event period is protracted (2). In the context of ongoing public health crises, like the COVID-19 pandemic, quality information is key for mobilizing the public to engage in self- and community-protective behaviors in the short and long terms. However, the pandemic response was complicated by mixed messaging from public health experts as the science evolved, and by local and federal government officials who disagreed on mitigation strategies to curb the rise of infections and deaths. Moreover, the expansive array of mediated information channels (e.g., news media, social media, and podcasts)—defined as a “conveyance device that collects information from a source or sources, repackages it and then disseminates it” (5)—at the public’s fingertips may have disadvantaged peoples’ ability to effectively cope psychologically and mobilize appropriately, especially if the messaging about the severity or nature of the threat differs among information channels that members of the public consult.

Trauma psychologists have long recognized the importance of the news media in collective trauma contexts (e.g., terror attacks and natural disasters), even during a pandemic (6)—not only do news media inform the public, but they can also be implicated in extending the psychological reach of a such events through repeated graphic coverage (7). A growing body of research demonstrates that total hours of daily media use is associated with negative psychological outcomes (for an overview, see ref. 8). However, the focus on hours of engagement across the vast media landscape fails to address other aspects of media use that may impact psychological well-being. For example, communications scholars have established a more sophisticated way of understanding media use, developing an approach that goes beyond time spent on various mediated channels to capture the general composition of the channels people attend to in daily life (9). This approach presents an opportunity for a more nuanced understanding of the role information channels play in how the public responds when collective traumas arise. Specifically, the COVID-19 pandemic offers a unique context in which to study the potentially consequential role of such channels in impacting psychological well-being and behavior during a protracted public health crisis.

The news media are collectively only one set of mediated information channels by which people acquire information during a crisis. The information channels on which people rely are spread across a dense web and continually shifting landscape of mediated information channels. While some individuals rely exclusively on conservative or liberal news, others may rely on both types of news media. Others still may be news avoidant, relying only on what they hear from neighbors or relatives [i.e., interpersonal information channels (2, 5)]; or they might exclusively attend to social media platforms for their news (2, 10, 11). The vast array of information channels available to us in any given time or context yields many idiosyncratic combinations of using these channels to serve our information needs. Communications scholars refer to such patterns as repertoires (9). Repertoires are operationalized in different ways within the communications space, including news repertoires that capture medium-specific patterns like television and print news media vs. online news (12) and information-specific patterns based on audience interest [e.g., “sports repertoire” (13)]. Much of this work explicates media use patterns free of any specific context (for an example, see ref. 14) in an effort to understand patterns of general media consumption and characterize the individuals exhibiting those patterns. However, rarely are these idiosyncratic patterns linked to specific outcomes. One notable exception of a repertoire approach employed with a vast trove of consumer cable channel use reveals how politically oriented repertoires are associated with county-level voter turnout (15).

In the context of a collective trauma, the composition of a repertoire is important because it may signal latent information about the types of channels that comprise it (e.g., opinions of pundits vs. professional journalists), as well as the content. A common theme in repertoire studies is the emergence of politically oriented repertoires, which may be particularly relevant in the context of the COVID-19 pandemic. It is now clear that the news media across the American political spectrum pushed different narratives about the COVID-19 threat, resulting in differential threat perceptions among members of the public. Evidence from a study of misinformation on prominent American news media channels in early March 2020 found that conservative news outlets were more likely to mention conspiracy theories about the virus’s origins compared with liberal news media outlets (16, 17). This work found that viewers of conservative news outlets were also more likely to believe that a vaccine already existed and were less likely to trust the CDC’s claims of the pandemic’s threat to public safety. Moreover, a Pew report in April 2020 (18) found that over half (56%) of respondents who attended to Fox News believed that the news media exaggerated the threat compared with 25% of CNN and 12% of MSNBC viewers; roughly a third of conservative viewers believed the virus was developed intentionally and reported seeing conflicting facts about the virus, respectively, compared with smaller proportions among liberal news viewers.

Such disparate framing across news media is likely associated with divergent psychological responses among viewers. Two decades of research has explicated the cross-sectional and longitudinal link between media exposure in the aftermath of collective traumas and psychological distress (7, 8). However, individuals attending to certain news media outlets may exhibit less psychological distress if those outlets frame events as less threatening; we know that how one appraises a stressful event is consequential for how one responds(19). To date, the focus in this area has been on news acquired through traditional (e.g., newspapers, television, and radio) and new media (e.g., online news and social media) in aggregate. In collective trauma contexts with disparate messaging, however, specific patterns of information-channel use may provide additional underexplored details and may shed light on psychological and behavioral responses.

Likewise, behavioral outcomes may also differ in marked ways depending on patterns of information-channel use. If, for example, people are led to believe a pandemic is not a credible threat, or at the very least is being unnecessarily exaggerated by the media, they may also be less likely to engage in health-protective behaviors (and engage in risky ones). Some evidence for this effect has been documented in the first year of the COVID-19 pandemic. Researchers found that county-level viewership of politically conservative news outlets was associated with decreases in physical distancing (which was a COVID-19 mitigation strategy encouraged by public health authorities) (20). This effect has also been documented in working papers by the National Bureau of Economic Research, collectively finding that county-level politically conservative news viewership was associated with decreases in compliance with stay-at-home orders (21) and increases in COVID-related cases and deaths (16).

The saturated media environment and differential framing across the landscape of information channels that individuals consulted during the pandemic may be consequential to psychological well-being and public health. In the current longitudinal investigation, we expand on the extant literature in communications and work at the intersection of media and trauma psychology by a) employing the concept of repertoires as a springboard from which to study dimensions of information channels used at the onset of the COVID-19 pandemic and b) to examine the demographic correlates of these dimensions. We then examined the prospective associations information-channel dimensions had with markers of psychological distress, COVID-related attitudes, and self-protective and risk-taking behaviors during this protracted event by following a large sample over time.

To address these issues, we used a probability-based, nationally representative sample of 5,661 Americans from the NORC AmeriSpeak panel who completed a survey beginning March 18, 2020 across the next 30 d (wave 1; N = 6,514) and again between September 26 to October 16, 2020 (wave 2; N = 5,661). This survey measured relevant distress-related, cognitive, and behavioral outcomes including COVID-related worry, global distress, emotional exhaustion, belief in the seriousness of the COVID-19 threat, confidence in one’s ability to protect oneself from COVID-19 (i.e., response efficacy), dismissive attitudes toward COVID-19, frequency of engaging in health-protective behaviors (e.g., wearing a mask, washing hands for 20 s), and engaging in risk-taking behaviors (e.g., getting on an airplane, going to a bar).

The survey also captured the top three information channels that respondents used in the past week to obtain COVID-related news. Additionally, NORC maintains demographic data on their panel members; thus, we had demographic information for each respondent (i.e., gender, age, race/ethnicity, education, and region of country), as well as prior doctor-diagnosed mental health and physical health ailments (measured before January 2020). The survey assessed political party identification, daily hours of COVID-related media exposure in the past week, direct exposure to COVID-19 (e.g., job requires in-person interaction, diagnosed with COVID-19), and secondary stressors due to the pandemic (e.g., lost job or wages, unable to find childcare; see ref. 22).

Missing data across these variables were accounted for using the full-information maximum likelihood estimation via the structural equation modeling command in Stata 16.1; the final sample consisted of 5,661 respondents (see *SI Appendix*, Table S1 for descriptive statistics for the sample compared against US census benchmarks). All reported ordinary least squares regression analyses were weighted to reflect population-level estimates, and estimates were standardized to allow for effect size comparisons among predictors.

## Deriving Information-Channel Dimensions

At wave 1, respondents indicated their top three COVID-related news information channels from a list of 31 channels (e.g., FOX News, CNN, and MSNBC). If an information channel was not listed, respondents had the opportunity to type it into a textbox. These text responses were coded and grouped based on eight commonly reported information channels including local news (e.g., television, radio, and newspapers), international news outlets, news aggregators (e.g., Google News and Apple News), social media (e.g., Facebook), government officials (e.g., Governors, the CDC), friends/family, far-right media (e.g., Newsmax and Breitbart), and other news media. This procedure produced a matrix of dichotomously coded information channel variables.

Respondents mostly indicated using mainstream news sources including Fox News (*n* = 2,255; 34.6%), ABC News (*n* = 2,173; 33.3%), CNN (*n* = 2,116; 32.4%), CBS News (*n* = 1,646; 25.2%), MSNBC (*n* = 1,158; 17.7%), The New York Times (*n* = 790; 12.1%), NPR (*n* = 705; 10.8%), and The Washington Post (*n* = 600; 9.2%). The remaining channels were each used by less than 9% of respondents. Additionally, a full third of our sample reported using fewer than three channels for COVID-related information. This matrix was submitted to a multiple correspondence analysis (MCA) to derive underlying patterns of information-channel use in the sample (a more detailed description of these procedures is contained in the *Methods*, below). The results of the MCA solution yielded four dimensions, representing distinct patterns of media exposure. The specific representative news channels, relevant literature and online resources, as well as discussions with a large social science lab, guided the labeling of each dimension: journalistic complexity, where higher scores are indicative of a focus on news with long articles and semantically and syntactically complex language (23) vs. basic, conversation-style reporting of news (24), ideological focus [right- vs. left-leaning news outlets; (25)], domestic focus (US- vs. international news outlets), and nonnews sources (Table 1). Representative information channels for each dimension are illustrated in *SI Appendix*, Figs. S1 and S2. Having derived information-channel dimensions, we then used the overall MCA solution to compute dimensional scores for each respondent [much like is possible with a principal component analysis (PCA)]. For example, a respondent who used both far right-leaning media and Fox News received a score of 0.54 on Dimension 2, whereas a respondent who used only far right-leaning information channels received a lower score of 0.40 and a respondent who used only Fox News received a score of 0.18. In contrast, a respondent who used CNN and Fox News received a score of −0.03 on Dimension 2. Thus, each respondent had a unique score along each dimension representing their *idiosyncratic* pattern of information-channel use.

**Table 1.** Descriptions of information-channel dimensions

Dimension	Description	Negative values	Positive values
1	Journalistic complexity	ABC News and CBS (less complex)	New York Times, NPR, and Washington Post (more complex)
2	Ideologically focused news	CNN and MSNBC (liberal-leaning)	Fox News and Far-right media (conservative-leaning)
3	Domestically focused news	PBS, BBC, and Reuters (globally centered)	USA Today (American-centered)
4	Nonnews		State or federal officials on television, proximal people, and social media

## Demographic Correlates of Information-Channel Dimensions

Respondents’ scores along each dimension were regressed onto demographic variables and key covariates to determine correlates of each information channel dimension (for a table of weighted means by demographics for each dimension, see *SI Appendix*, Table S1). Specifically, these analyses determined whether there were 1) significant demographic predictors of information-channel dimensions and 2) significant prospective associations between these dimensions and measures of psychological well-being, cognition, and behavior, respectively.

### Journalistic Complexity.

Women and Black respondents exhibited lower journalistic complexity scores relative to men and White respondents, respectively. Relative to younger adults (aged 18 to 25), older adults had significantly lower journalistic complexity scores, suggesting they reported relying on information channels (e.g., ABC and CBS) characterized by basic reporting of news. More educated respondents had significantly higher scores on journalistic complexity relative to less educated respondents, suggesting they indicated relying on complex reporting (e.g., long articles with in-depth analysis; The New York Times, Washington Post). Respondents who reported fewer daily hours of media engagement exhibited significantly higher complexity scores than those with more daily hours. More politically conservative respondents exhibited significantly lower complexity scores than their liberal-leaning counterparts.

### Ideological Focus.

Women compared with men, and respondents 60+ y old compared with younger adults, exhibited significantly lower ideological focus scores, suggesting that on average women and older people attended more to liberal-leaning news channels (e.g., CNN and MSNBC). Respondents reporting more daily hours of media engagement exhibited significantly lower scores on this dimension as well. Also, respondents with a conservative-leaning party identity exhibited significantly higher ideological focus scores, suggesting that they attended to right-leaning news channels (e.g., FOX News and Breitbart).

### Domestic Focus.

Relative to younger respondents, all other age groups exhibited lower domestic focus scores, suggesting that they are more reliant on international news channels (e.g., PBS, BBC, and Reuters). Results also suggest that respondents reporting increased daily hours of media engagement and a conservative-leaning party identity exhibited higher domestic focus scores, suggesting that they attended to generally American-focused news (e.g., USA Today).

### Nonnews.

Relative to men, women exhibited higher nonnews scores, suggesting that they relied on people in their lives, social media, and experts on television more than news channels to get information about the pandemic. Older adults (60+) exhibited lower nonnews scores. Respondents with increased daily hours of media engagement exhibited lower scores on this dimension.

### Political Party Identification.

**Fig. 1.**
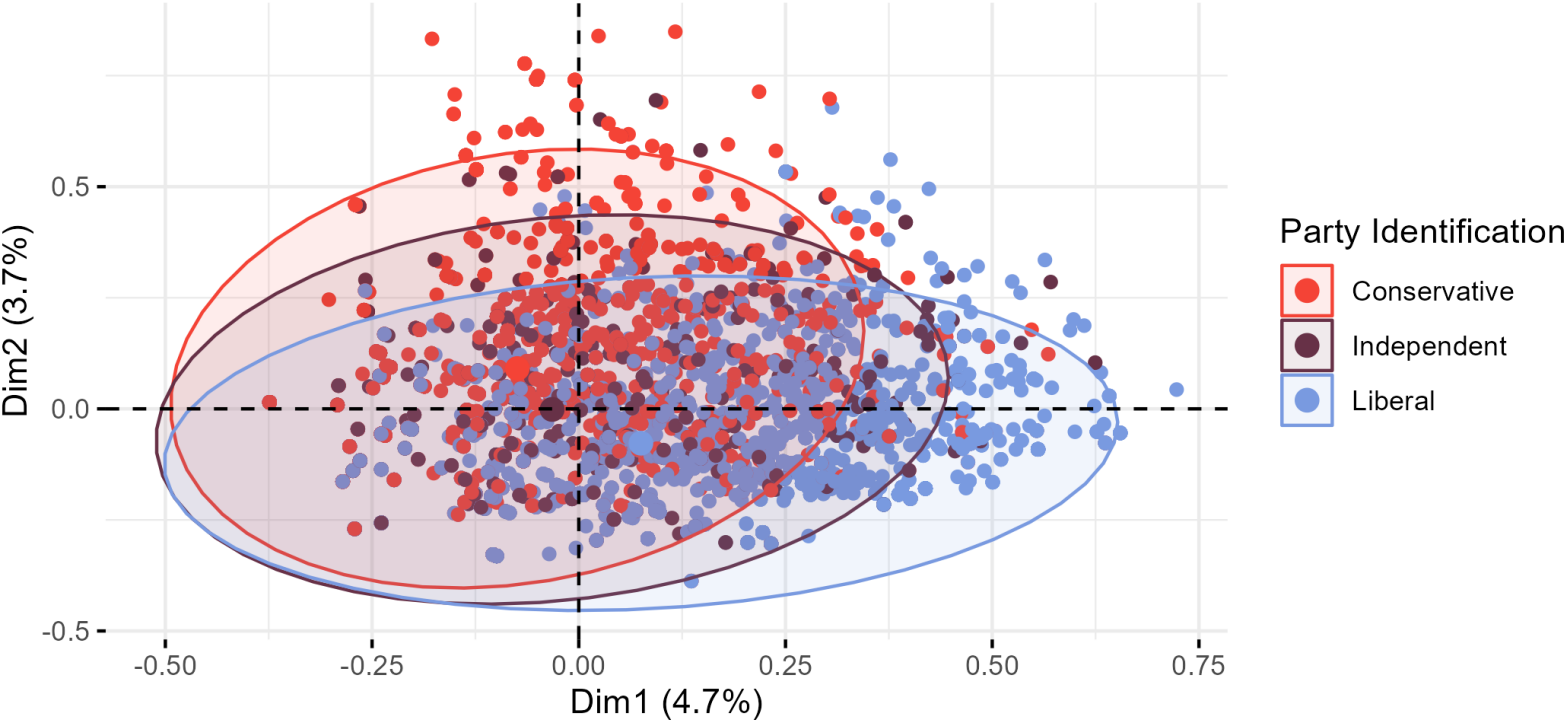
Individual-level plot of respondents, colored by their political party identification, in multidimensional space based on their information-channel dimension scores along dimensions 1 (journalistic complexity) and 2 (ideological focus). Ellipses are drawn around each group, providing detail about the breadth and shape of the distributions for each group.

Given that political party identity was a strong correlate across the first two information-channel dimensions (journalistic complexity and ideological focus), we evaluated the distribution of respondents in multidimensional space by their party identification. Respondents were grouped by their ideological orientation (i.e., conservative, independent, or liberal) and colored accordingly (Fig. 1). The software also drew ellipses around each group to provide clarity around where the bulk of respondents from each group resided in dimensional space. The conservative ellipsis fell higher along the ideological focus dimension relative to the liberal ellipsis (which fell lower on the axis). The ellipsis for independents was more centered. What is noteworthy about this representation is that although the breadth and shape of each ellipse fell in line with expectations, many respondents from across the political spectrum had scores along ideological focus that were not in line with their party identity. For example, several red dots appear in a more liberal-leaning space and several blue dots appear in a more conservative space. This suggests that political party identification is not a sure-fire marker of selective news exposure, as others have noted (15, 26).

## Prospective Associations between Information-Channel Dimensions and Distress, Cognition, and Behavior

**Fig. 2.**
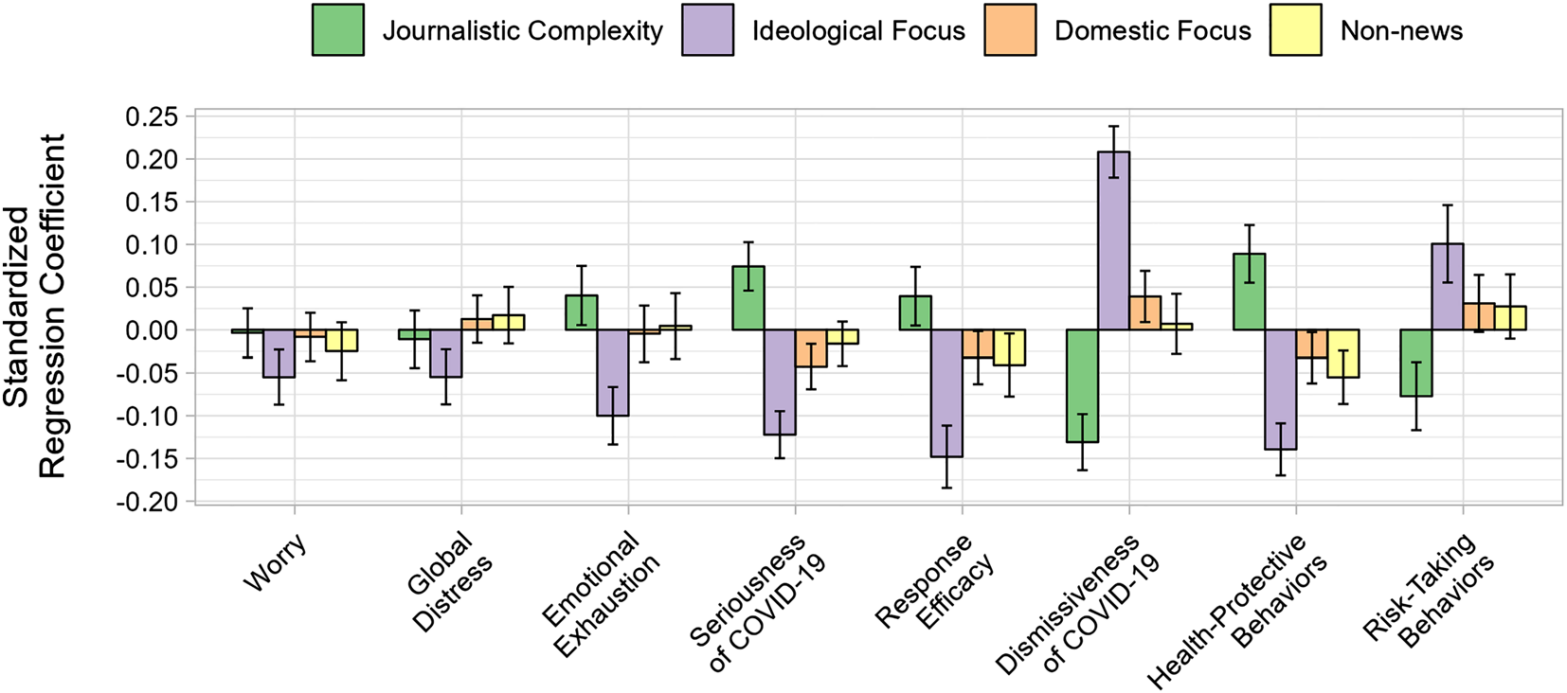
Standardized regression coefficients and CIs for information-channel dimensions across eight outcomes. All dependent variables were measured at wave 2 and control for wave 1 responses (where applicable). All analyses also control for demographic variables (i.e., gender, age, race/ethnicity, education, and region), prior doctor-diagnosed mental health and physical health ailments each measured before the COVID-19 study began, a continuous measure of political party identification (Strong Democrat to Strong Republican), daily hours of COVID-related media exposure in the past week, direct exposure to COVID-19 (e.g., job requires in-person interaction, diagnosed with COVID-19), and secondary stressors (e.g., lost job or wages, unable to find childcare). Note: Positive values of journalistic complexity represent more reliance on complex news content; Positive values of ideological focus represent more reliance on conservative-leaning news channels; Positive values of domestic focus represents more reliance on American-centered news; Positive values of nonnews channel use represent a reliance for information about COVID-19 on social media, people such as neighbors and spouses, and federal and state officials seen on television.

We next evaluated the prospective associations idiosyncratic information-channel dimensions (measured at wave 1) had with each outcome variable (measured 6 mo later at wave 2). Each outcome was simultaneously regressed onto all dimensions as well as relevant covariates in a series of weighted and standardized ordinary least squares regression models. Where applicable, analyses controlled for wave 1 measurements of outcome variables. Results for these standardized models are depicted in Fig. 2 (full tables of results are available in supplement; see *SI Appendix*, Tables S2–S4).

### Psychological Well-Being.

Results indicated that respondents favoring liberal-leaning news reported more COVID-related worry compared with respondents favoring conservative-leaning news (*b* = −0.05, *SE* = 0.01, *P* = 0.001). A similar pattern was evident for global distress (*b* = −0.05, *SE* = 0.01, *P* = 0.001) and emotional exhaustion (*b* = −0.10, *SE* = 0.01, *P* < 0.001). Additionally, respondents exhibiting higher journalistic complexity scores reported more emotional exhaustion (*b* = 0.03, *SE* = 0.01, *P* = 0.02) relative to those who indicated relying on basic news reporting.

### Cognition.

The associations between information-channel dimensions appeared to be stronger for attitudes when compared with the markers of psychological well-being, as evidenced by the larger standardized regression coefficients. Belief in the seriousness of COVID-19 was associated with more journalistically complex (*b* = 0.07, *SE* = 0.01, *P* < 0.001), liberal-leaning (*b* = −0.12, *SE* = 0.01, *P* < 0.001), and internationally focused (*b* = −0.04, *SE* = 0.01, *P* = 0.002) patterns of information channel use, respectively.

A similar pattern across dimensions was observed for response efficacy (journalistic complexity: *b* = 0.04, *SE* = 0.01, *P* = 0.02; ideological focus: *b* = −0.14, *SE* = 0.01, *P* < 0.001; international focus: *b* = −0.03, *SE* = 0.01, *P* = 0.04), suggesting that confidence in one’s ability to protect oneself was associated with more journalistic complexity, liberal leaning media, and internationally focused news, respectively. Individuals who indicated getting their information primarily by relying on people in their lives, social media, and from experts on television reported lower response efficacy than those who attended to news channels (*b* = −0.04, *SE* = 0.01, *P* = 0.03).

Results also suggested that less journalistic complexity (*b* = −0.13, *SE* = 0.01, *P* < 0.001), attending more to right-leaning media (*b* = 0.20, *SE* = 0.01, *P* < 0.001), and domestically focused news (*b* = 0.04, *SE* = 0.01, *P* = 0.01) were each associated with more dismissive attitudes toward the COVID-19 threat, respectively.

### Behavior.

Results also indicated that increasing journalistic complexity was associated with engaging in significantly more health-protective behaviors (*b* = 0.08, *SE* = 0.01, *P* < 0.001). Conversely, attending more to right-leaning media was associated with engaging in significantly fewer health-protective behaviors (*b* = −0.13, *SE* = 0.01, *P* < 0.001). Likewise, those attending to more domestically focused news (*b* = −0.03, *SE* = 0.01, *P* = 0.03) and those who primarily relied on people, social media, and experts on television (*b* = −0.05, *SE* = 0.01, *P* < 0.001), reported engaging in significantly fewer health-protective behaviors.

More journalistic complexity was also associated with significantly fewer risk-taking behaviors (*b* = −0.07, *SE* = 0.02, *P* < 0.001). Attending to more right-leaning channels was significantly associated with more risk-taking behaviors (*b* = 0.10, *SE* = 0.02, *P* < 0.001).

## Discussion

This large longitudinal investigation reveals that in the context of a collective trauma like the COVID-19 pandemic, people idiosyncratically rely on collections of specific information channels to stay informed about the crisis. Respondents in our sample exhibited patterns of information-channel use falling along four dimensions representing journalistic complexity, ideologically focused news, domestically focused news, and nonnews information channels. Moreover, respondent scores along these dimensions early in the pandemic were prospectively associated with markers of psychological well-being, COVID-related attitudes, and health-protective and risk-taking behaviors measured 6 mo later. Our results suggest that the information channels with which people engage during a protracted crisis may be consequential for public health.

The patterns of information-channel use we identified are conceptually similar to those found in other work linking news repertoires to important outcomes. For example, using a large database of cable news channels used by over 160k households in the United States, others have identified news consumption patterns that fell along varying degrees of political ideology (15). Additional patterns found in this work included those among participants dubbed newshounds and news avoiders, or households that consume more news than others and those that use far less, respectively (15).

Our approach in this work extends prior studies at the intersection of trauma and media psychology and takes inspiration from research on media repertoires in the communications literature. Rather than statistically linking either total media exposure or each specific information channel respondents used to outcomes of interest, which is common in other related work (2, 7, 8, 10), we instead derived patterns of information-channel use based on the sources respondents indicated relying on to get news about the COVID-19 pandemic. Specifically, our approach allowed us to compute targeted, idiosyncratic scores for each respondent along four dimensions of information-channel use and then use these scores to prospectively predict outcomes.

After controlling for relevant covariates (including respondent political ideology), the patterns of information-channel use we identified were prospectively associated with psychological, cognitive, and behavioral outcomes measured 6 mo later in meaningful ways. For example, we found that patterns of channel use characterized by journalistic complexity was associated with more emotional exhaustion, belief in the seriousness of COVID-19, greater response efficacy, and engaging in health-protective behaviors. This pattern of response is notable given higher scores on this dimension were associated with higher education, a liberal party identity, and younger age. As others have noted, the motivation to use specific information channels hinges in part on perceived usefulness and relevance of those channels to inform its audience of the crisis (27). Additionally, whether a channel is used also depends on how quickly consumers can evaluate an imminent threat based on the information presented and the time they have with which to engage that information (27). In the context of the COVID-19 pandemic, an imminent and protracted threat, a reliance on complex reporting was possible and informative, allowing for individuals with the access, education, and time to understand the full impact of the threat and how to take action to protect themselves and others. This supposition is consistent with past work showing that systematic (or deeper) processing of risk information (necessary if one reads nuanced, in-depth news articles) increases health-protective behaviors (28).

Given the differential partisan framing of the COVID-19 threat, it is perhaps unsurprising that respondents who relied on conservative-leaning news reported less psychological distress than respondents who relied on liberal-leaning news. Conservative news media downplayed the threat of COVID-19 from the onset, and while that had community-level consequences (16), our data show that it was also linked to attenuated threat appraisals—attending to conservative-leaning news was associated with less belief in the seriousness of the threat, less response efficacy, and more dismissive attitudes over time. We also found that attending to conservative-leaning news was associated with engaging in fewer health-protective behaviors and more risk-taking behaviors, relative to respondents who were attending to liberal-leaning news. Our findings add to a growing body of evidence highlighting the importance of news media framing during the COVID-19 pandemic (16, 21), but also reveal the role the news media have in not only informing the public during protracted health crises, but also the consequences of that information for influencing public health.

Our analysis also identified a pattern of information-channel use characterized by internationally vs. domestically focused (American) news. Domestically focused news consumption was associated with more dismissive attitudes toward the COVID-19 threat, less belief in the seriousness of the COVID-19 threat, lower response efficacy, and engaging in fewer protective health behaviors relative to the consumption of internationally focused news. Given that the COVID-19 spread all over the world, respondents who attended to international news were likely exposed to reports confirming that the threat posed a real and present danger to personal and community health. In contrast, American-focused news was likely flooded with conflicting information and the controversy of the pandemic response, in addition to reports of the pandemic’s impact. This diversity of information may have activated a more heuristic processing of threat (27) and interfered with respondents’ threat appraisals and sense of response efficacy.

The inclusion of an open-ended item to capture additional information channels respondents relied on for COVID-related information allowed for a data-driven analysis of nontraditional sources of news (i.e., nonnews channels). Our analyses revealed that some respondents avoided the news altogether and relied solely on other people in their immediate off- and online social networks [e.g., interpersonal channels (27)], as well as press conferences by government officials and medical experts for COVID-related information. Despite health officials being included in this dimension, higher scores were associated with less response efficacy and engaging in fewer health-protective behaviors. It may be the case that news conferences with experts addressing the public also featured political leaders, whose statements were at odds with the health-protective recommendations being suggested. Additionally, a reliance on social media, which has been documented as a source of misinformation during the pandemic (29), may have misled users about the severity of the threat, thus attenuating confidence in the efficacy of and actual engagement in health-protective measures.

We acknowledge some limitations of this work. First, we asked our respondents to indicate the top three information channels they used for COVID-related news. Undoubtedly, many respondents used more than three, and the list we provided was not comprehensive enough to capture cross-media channels (except for cases in which respondents typed in “social media,” named a social media platform, or named a local television or radio station in the open-ended text field). Additionally, we did not ask respondents the extent to which they relied on each channel they reported. There is evidence that individuals are not adept at reporting their media use over time (30); however, reporting a more general assessment of the most commonly used media channels for COVID-related information is likely an easier task for participants to complete accurately. It is also worth noting that we did not assess the extent to which respondents trusted the channels they relied on for COVID-related information. It could very well be the case that respondents whose patterns of information-channel use spanned the ideological spectrum, for example, attended to channels they tend to not trust to gauge how the “other side” framed the pandemic. To account for this, future MCA procedures should be weighted to include reliance on and trust in each information channel.

It is also not clear whether the patterns we identified are consistent with respondents’ usual daily news media diets, or if they arose specifically in response to the COVID-19 threat. Ambiguous crisis situations often lead individuals to engage in information-seeking behavior from new and/or different information channels (31) to acquire information and thus mitigate psychological discomfort with uncertainty. Although the patterns we identified are consistent with other studies of general news media patterns, it remains an open question whether crisis-specific patterns differ from general ones. Additionally, we assumed that the information channels people consulted at the onset of the pandemic remained consistent through the follow-up survey. However, it is possible patterns of media consumption changed over time as the crisis unfolded. Future studies should evaluate the extent to which patterns of information-channel use shift over time to fully characterize the media frames to which individuals may have been exposed.

Despite these limitations, the patterns we identified offered utility in predicting important markers of psychological well-being, cognition, and behavior 6 mo later. This work expands on the extant literature at the intersection of media and trauma psychology in two distinct ways. First, rather than surveying different collections of information channels (e.g., television and radio), we focused exclusively on capturing the specific news channels respondents used to get updates about the pandemic. This allowed us to understand the types of news displays and content to which respondents were likely exposed. Second, we also provided a space for respondents to indicate other communication channels through which they obtained COVID-related updates (e.g., other people, social media). The advantage of this approach is that it offered granularity in characterizing the specific sources of information (besides standard news channels), driving the fourth dimension we identified.

## Conclusion

Taken in sum, our findings add a layer of complexity to past analyses of the link between news media exposure in the context of adversity and collective traumas; time spent engaging with news media is but one important aspect of exposure. We find that collective traumas may be appraised differently based on the information channels members of the public use to gain information. Thus, the type of channels with which people engage, as well as news media framing of protracted threats, may be consequential for psychological well-being and public health. We believe that our analyses suggest that diversifying the information channels we rely on for critical updates is important for developing and maintaining a well-rounded perspective on an ongoing crisis. Moreover, understanding aspects of information channels people use during such events may be useful for explicating how the public responds psychologically and behaviorally to collective threats.

## Methods

### Study Sample and Procedure.

Respondents for this multiwave study were drawn from the NORC AmeriSpeak Panel, a probability-based panel of 35,000 US households who have been selected at random from across the United States to create a representative sample of US adults. The AmeriSpeak panel is the only probability panel in the United States that uses random door-to-door interviewing to recruit its participants, who subsequently participate in AmeriSpeak surveys via the web. As a result, AmeriSpeak attains response rates nearly three times higher than any other probability panel in the United States (32). Unlike typical Internet panels, in which people who already have Internet access can choose to opt in, no one can volunteer for the AmeriSpeak panel.

The wave 1 survey was fielded to a sample of 11,139 panelists beginning on March 18, 2020 (5 d after the US President’s declaration of a national emergency) in three cohorts of 10 d fielding periods and continued until April 18, 2020 (22). Most respondents (86.4%) completed the survey within the first three days of data collection. Participants received an email stating that the survey was available. Surveys were confidential, self-administered, and accessible any time for a designated period; participants could complete it only once. Almost 44% completed the survey on a computer, about 54% completed it on a smartphone, and the remainder completed it on a tablet (or did not provide a response). NORC compensated AmeriSpeak panelists with points worth a cash equivalent (in this case $4). When the fielding period ended, 6,598 had completed surveys (59.2% completion rate); 84 cases (1.3%) were removed from the final sample due to unreliable survey completion times (under 6.5 min) or extensive missing data (>50% of questions), leaving N = 6,514 panelists (58.5% completion rate).

The wave 2 survey was fielded 6 mo later (September 26 to October 16, 2020) to all available wave 1 participants (6,501 panelists). Of these, 5,661 completed the wave 2 survey (87.1% completion rate). Most respondents (80.1%) completed the survey within the first four days of data collection.

Participants provided informed consent when they joined the NORC panel and were informed that their identities would remain confidential. All procedures for this study were approved by the Institutional Review Board of University of California, Irvine.

### Dependent Variables.

The surveys evaluated several constructs related to distress, cognition, and behavior related to the COVID-19 pandemic. Distress-related dependent variables include COVID-related worry, global distress, and emotional exhaustion. Cognition-related dependent variables included belief in the seriousness of COVID-19, response efficacy, and dismissive attitudes about COVID-19. Behavior-related dependent variables included engaging in self-protective and risk-taking behaviors, respectively. Specific details about each measure are presented below.

### Distress Variables.

#### COVID-related worry (W1 & W2).

At each wave, respondents completed a 10-item index of COVID-related worries adapted from measures used in prior research (33, 34). Items measured worries in the previous week about COVID-19 affecting both participants and their loved ones. Example items included worries and fears about not acquiring basic necessities, struggling to get healthcare, not having enough money to pay bills, civil unrest, or getting sick and dying from the Coronavirus. At wave 2, the measure was modified slightly such that the items about acquiring basic necessities were replaced with items measuring worry about exposure to COVID-19 when leaving the house. At each wave, items were assessed on a 1 (*Never*) to 5 (*All the time*) Likert-type scale and averaged into a composite variable at each wave; measure reliability was excellent (wave 1 α = 0.91; wave 2 α = 0.91).

#### Global distress (W1 & W2).

Respondents completed assessments of global distress symptoms using the Brief Symptom Inventory-18 [BSI-18; (35)] across waves. The BSI measured symptoms associated with somatization, anxiety, and depression via 18 items (six items for each subscale); an additional three items measuring anger experienced in the past week were also assessed. In wave 1, this 21-item measure was administered, and in wave 2, a shorter 9-item version of the BSI that has been used in previous studies (7) was administered to the sample along with the three additional wave 1 anger items. Responses across each symptom ranged from 0 (*Not at all*) to 4 (*Extremely*). Scale reliability was excellent at wave 1 (α = 0.92) and wave 2 (α = 0.89). Responses across items were averaged to create a composite score at each wave.

#### Emotional exhaustion (W2).

At wave 2, respondents answered a 6-item measure related to how often they have felt emotionally exhausted, overwhelmed, and stressed in the past week. Responses ranged from 1 (*Never*) to 5 (*All the time*). Three items were reverse scored (i.e., coping well; felt hopeful about the future; in control). The scale was reliable (α = 0.86). Responses across items were averaged to create a composite score.

### Cognitive Variables.

#### Belief in the seriousness of COVID-19 (W1 & W2).

Across both waves of data collection, respondents indicated the extent to which they agreed with statements pertaining to the seriousness of COVID-19. Example items include “Coronavirus is a serious threat to our society”, “Coronavirus is more contagious than the flu”, and “The government is exaggerating the Coronavirus threat” (reverse scored). Responses ranged from 1 (*Strongly Disagree*) to 5 (*Strongly Agree*). Scale reliability was good at wave 1 (α = 0.81) and wave 2 (α = 0.89). Responses across items were averaged to create a composite score at each wave.

#### Response efficacy (W1 & W2).

Across both waves, respondents rated their agreement with items assessing their confidence in their ability to protect themselves from COVID-19. At wave 1, this measure included 2 items measuring confidence that COVID-mitigation efforts (e.g., social distancing and increasing hand hygiene) would protect them from the virus. At wave 2, this list of items was updated with two additional items to reflect response efficacy with respect to wearing masks to protect the self and others, respectively. Responses for each wave ranged from 1 (*Strongly Disagree*) to 5 (*Strongly Agree*). Scale reliability was good at wave 1 (α = 0.78) and wave 2 (α = 0.87). Responses across items were averaged to create a composite score at each wave.

#### Dismissive attitudes (W2).

At wave 2, respondents reported their agreement with five items about their beliefs about their personal ability to resist a COVID-19 infection. Example items included “My immune system can fight COVID-19” and “Getting COVID-19 is not dangerous for someone like me.” Responses ranged from 1 (*Strongly Disagree*) to 5 (*Strongly Agree*). Scale reliability was good (α = 0.79). Responses across items were averaged to create a composite score.

### Behavior Variables.

#### Health-protective behaviors (W1 & W2).

Across both waves, respondents were given items assessing how often they engaged in health-protective behaviors. At wave 1, this measure included eight items (e.g., wash my hands/use hand sanitizer more often, wear a face mask and/or gloves in public, avoid public transportation). At wave 2, items were updated to reflect the Centers for Disease Control (CDC) recommended practices at the time the survey was administered. Six items were used to measure health-protective behaviors and included items like “wash my hands for at least 20 s”, “wear a face mask in public”, “avoid nonessential personal care services (e.g., medical/dental visits, haircuts/barbers).” Responses ranged from 1 (*Never*) to 5 (*All the time*). Scale reliability was good at wave 1 (α = 0.76) and wave 2 (α = 0.79). Responses across items were averaged to create a composite score at each wave.

#### Risk-taking behaviors (W2).

Respondents answered a series of eight questions about their activities since COVID-19 restrictions were relaxed in their communities. Example items included “flown on an airplane”, “gone to a bar”, “worked out at a gym or fitness studio (indoors)”. Responses ranged from 0 (no) to 2 (more than once). Responses across items were summed to create a composite score, with higher scores indicating greater risk-taking behaviors.

### Independent Variables.

#### Information-channel dimensions.

At wave 1, respondents were asked to select (via a checkbox) their top three news media channels for COVID-related information from a list of 31 channels. Responses were dichotomous indicators of using each channel (1 used; 0 not used). The list included major cable (e.g., FOX News, CNN, and MSNBC) and commercial broadcast (e.g., ABC and CBS news) network news, international news agencies (e.g., Associated Press, Reuters, and BBC), public television (e.g., PBS), as well as topically focused news sources (e.g., Vox, Forbes, and Wired). Respondents could also check “Other” and type into a text box any other information channels they relied on for COVID-related news. These open-ended responses were coded for their content using automated techniques capturing most frequent words and word pairs (i.e., bigrams), as well as human inspection of each entry. Nine additional information-channel groups were added, and respondents were coded dichotomously across these groups. They included: NBC (which was not listed among the original 31 news channels), local news outlets (local television and newspapers), social media platforms, people in their lives (e.g., spouse, children, and neighbors), news aggregators (e.g., Google News and Apple News), and right-wing news channels [e.g., Newsmax and Breitbart; (25)]. For example, if a respondent used either Newsmax or Breitbart, they were coded 1 (all others coded 0) on this right-wing news variable (Table 2).

**Table 2.** Standardized demographic differences and key correlates of information-channel dimensions (N = 5,661)

Variables	Journalistic complexity	Ideologically focused news	Domestic focused news	Nonnews
	*b*(*se*)	*b*(*se*)	*b*(*se*)	*b*(*se*)
Women (0 = men)	−0.07(0.01)***	−0.08(0.01)***	0.02(0.01)	0.10(0.02)***
Race/ethnicity (0 = White)				
Black/African American	−0.14(0.01)***	−0.03(0.01)	0.03(0.02)	−0.02(0.01)
Hispanic	−0.02(0.01)	0.01(0.01)	−0.001(0.01)	0.007(0.01)
Other	−0.04(0.02)	−0.01(0.02)	0.05(0.02)*	−0.03(0.02)
Age (0 = 18 to 29)				
30 to 44	−0.14(0.02)***	−0.05(0.02)*	−0.08(0.03)**	−0.01(0.03)
45 to 59	−0.25(0.02)***	−0.07(0.02)*	−0.10(0.03)**	−0.05(0.03)
60+	−0.30(0.02)***	−0.13(0.02)***	−0.17(0.03)***	−0.07(0.03)*
Education (0 = No HS diploma)				
HS diploma	0.01(0.04)	−0.03(0.04)	0.04(0.04)	−0.07(0.05)
Some college	0.13(0.04)***	−0.02(0.03)	0.05(0.04)	−0.09(0.05)
BA or above	0.33(0.04)***	−0.02(0.03)	0.03(0.04)	−0.11(0.05)
Region (0 = East)				
Midwest	−0.04(0.02)*	−0.04(0.02)*	0.001(0.02)	−0.01(0.02)
South	−0.03(0.02)	0.002(0.02)	−0.01(0.03)	−0.02(0.02)
West	0.03(0.02)	−0.02(0.02)	0.004(0.03)	−0.01(0.02)
Media exposure	−0.09(0.02)***	−0.05(0.02)*	0.12(0.02)***	−0.14(0.02)***
Political party identity	−0.26(0.01)***	0.36(0.01)***	0.06(0.01)***	−0.02(0.01)

Political party identity is coded such that lower values correspond to more identification with the Democratic Party and higher values correspond to more identification with the Republican Party. Note: *P < 0.05; **P < 0.01; ***P < 0.001.

This matrix of dichotomously coded information channel data was submitted to a MCA using the FactoMineR package (36) in R (37). MCA is a generalized form of PCA designed specifically for handling categorical data (38) and determines underlying response patterns across variables. A scree plot was generated to examine the proportion of variance explained by each MCA dimension. Although the percentage of variance explained was low for each individual dimension, the scree plot indicated that four dimensions was an appropriate solution. As others have noted (39, 40), variance explained, as a measure of the quality of the solution, is misleading in the case of binary data. However, our goal was not to explain variance but rather to use a machine-learning approach in an effort to parse and meaningfully describe patterns of information channel use.

### Analytic Strategy.

Statistical analyses were run in Stata 16.1 (StataCorp). A series of ordinary least squares regression analyses were conducted (one for each outcome) to determine the extent to which information-channel dimensions were prospectively associated with distress (i.e., worry, global distress, emotional exhaustion), cognition (i.e., beliefs about the seriousness of COVID-19, response efficacy, and dismissive attitudes), and behavior (i.e., engaging in health-protective behaviors, risk-taking behaviors) measured at wave 2 (controlling for wave 1 outcomes where applicable).

Although these analyses cannot determine causal relationships between variables, they reveal the extent to which patterns of information channel use were linked with outcomes 6 mo later, above and beyond the impact of outcome variables measured at the onset of the pandemic. All analyses were standardized and weighted to reflect population estimates.

#### Covariates.

Given that several factors are known to be associated with the outcomes we studied, relevant covariates were included in each analysis. In addition to demographic variables (i.e., gender, age, race/ethnicity, education, and region of residence), analyses controlled for prior mental and physical health diagnoses (measured before January 2020), political party identification measured continuously (1 – strong Democrat to 7 – strong Republican), daily hours of COVID-related media use in the past week, direct exposure to COVID-19 (e.g., job requires in-person interaction, diagnosed with COVID-19), and secondary stressors (e.g., lost job or wages, unable to find childcare; see ref. 22).

## Supplementary Material

Appendix 01 (PDF)

## Data Availability

Anonymized (Anonymized project data) data have been deposited in Open Science Framework (OSF) (41).
