# Supplementary material for: Idiosyncratic media exposures during a pandemic and their link to well-being, cognition, and behavior over time
Source: Proc Natl Acad Sci U S A. 2023 Jun 20;120(26):e2304550120. doi: 10.1073/pnas.2304550120 (PMC10293849; doi:10.1073/pnas.2304550120)

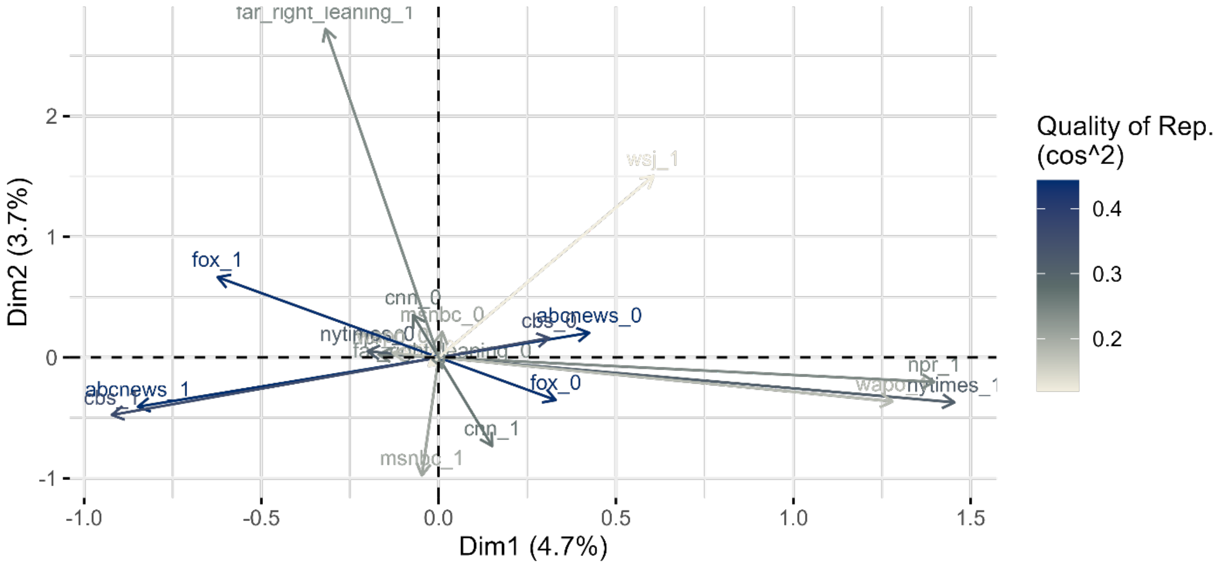

Figure S1. Dimensions 1 and 2 of a four-dimension MCA solution. Values along Dimension 1 (x axis) represent journalistic complexity of news channels. Negative values correspond with channels exhibiting short fact-based news reports and positive values with those exhibiting longer reports and complex analysis. Values along Dimension 2 (y axis) represent ideological slant of opinion-based news channels. Negative values correspond with liberal-leaning channels and positive values with conservative-leaning channels.

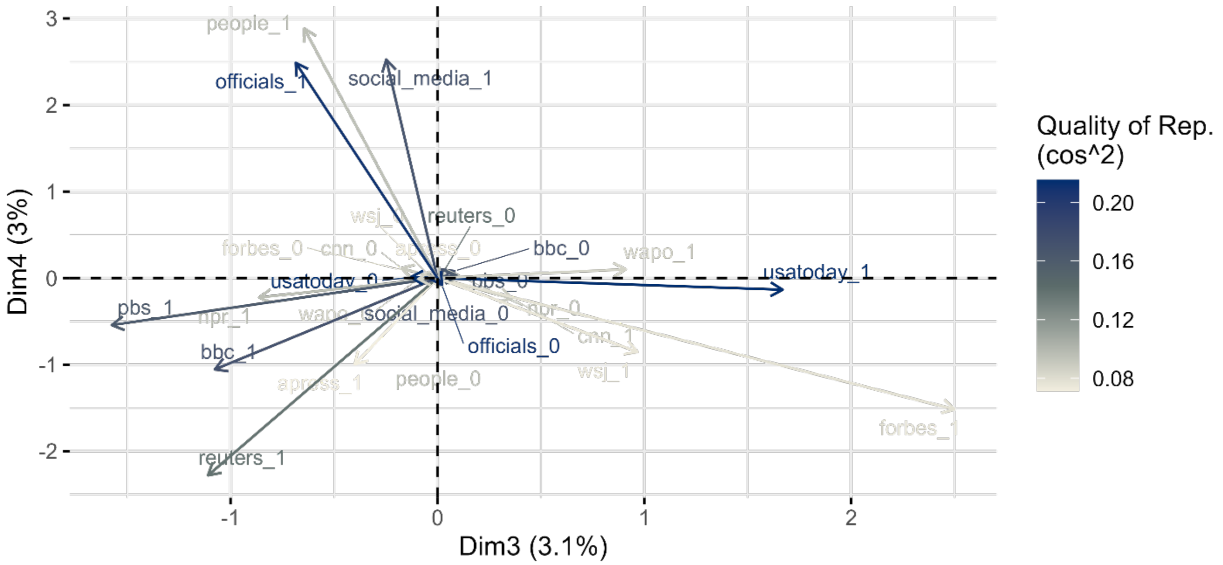

Figure S2. Dimensions 3 and 4 of a four-dimension MCA solution. Values along Dimension 3 (x axis) represent the domestic focus of news channels. Negative values correspond with channels focusing on international news and positive values on news channels with an American focus. Positive values along Dimension 4 (y axis) represent a reliance on non-news information channels.

Table S1. Descriptive statistics for dimension scores by demographic variables.

	Journalistic complexity		Ideological focus		Domestic vs. International focus		Non-news channels	
	M	SD	M	SD	M	SD	M	SD
Gender								
Male	0.003	0.220	0.023	0.202	0.000	0.187	-0.020	0.191
Female	-0.035	0.212	-0.020	0.176	0.010	0.165	0.014	0.162
Race/Ethnicity								
White	-0.010	0.218	0.017	0.193	-0.008	0.172	0.004	0.177
Black	-0.091	0.186	-0.069	0.148	0.017	0.154	-0.018	0.133
Other	0.025	0.218	0.001	0.212	0.022	0.176	-0.007	0.222
Hispanic	-0.012	0.220	-0.014	0.179	0.040	0.198	-0.011	0.181
Age								
18-29	0.066	0.223	0.018	0.179	0.049	0.217	0.010	0.218
30-44	0.007	0.218	0.002	0.172	0.011	0.175	0.005	0.190
45-59	-0.058	0.200	0.004	0.199	0.002	0.157	-0.008	0.162
60+	-0.060	0.203	-0.016	0.204	-0.028	0.151	-0.012	0.143
Education								
No HS diploma	-0.088	0.173	0.004	0.149	0.008	0.132	0.024	0.190
HS graduate	-0.089	0.186	0.001	0.182	0.012	0.173	-0.002	0.174
Some college	-0.028	0.202	0.003	0.200	0.011	0.180	-0.005	0.173
BA or above	0.074	0.230	-0.004	0.199	-0.006	0.185	-0.007	0.180
Region of residence								
Northeast	-0.002	0.232	0.000	0.178	0.004	0.179	0.003	0.165
Midwest	-0.034	0.202	-0.008	0.186	0.001	0.174	0.004	0.169
South	-0.042	0.209	0.006	0.194	0.006	0.169	-0.009	0.177
West	0.028	0.221	-0.001	0.196	0.008	0.184	0.000	0.193

Table S2. Standardized regression results for analyses across domains of emotion (N=5,661).

	**Worry**			**Global Distress**			**Emotional Exhaustion**		
Variables	*b*	*SE*	*p*	*b*	*SE*	*p*	*b*	*SE*	*p*
Journalistic Complexity	<.001	.01	.81	-.01	.02	.53	.04	.02	.02
Ideological Focus	-.01	.01	<.001	-.05	.02	<.001	-.10	.02	<.001
Domestic Focus	-.01	.01	.57	.01	.01	.37	<.001	.02	.79
Non-news Information Channels	-.02	.02	.15	.02	.02	.31	<.001	.02	.81
Woman (0 = Man)	.03	.01	.05	.02	.02	.89	<.001	.02	<.001
Race/Ethnicity (reference = White)									
Black/African American	.02	.02	.25	<.001	.02	.71	.13	.02	<.001
Other	.06	.01	<.001	-.01	.02	.07	-.08	.02	.90
Hispanic	.05	.02	.02	.03	.01	.32	<.001	.02	.46
Age (reference = 18-29)									
30-44	.03	.02	.18	.02	.02	.73	-.01	.02	.11
45-59	.04	.02	.04	.01	.02	.16	-.04	.02	<.001
60+	.10	.02	<.001	-.03	.02	.05	-.15	.02	<.001
Education (reference = No HS diploma)									
HS graduate or equivalent	.02	.04	.65	-.05	.03	.58	-.31	.03	.79
Some college	.02	.03	.46	-.02	.04	.18	-.01	.04	.05
BA and above	.02	.04	.60	-.05	.04	.15	-.07	.04	.08
Region (reference = Northeast)									
Midwest	.02	.02	.37	-.06	.04	.53	-.07	.04	.54
South	.03	.02	.21	.01	.02	.12	.01	.02	.24
West	<.001	.02	.84	.03	.02	.24	.03	.02	.87
Mental health diagnosis (0 = No diagnosis)	.05	.02	<.001	.02	.02	<.001	<.001	.02	<.001
Prior physical health ailments	.03	.01	.05	.13	.02	.30	.23	.02	<.001
Media exposure	.07	.02	<.001	.02	.02	<.001	.05	.02	<.001
Political party identification	-.11	.02	<.001	.07	.02	.08	.10	.02	<.001
Direct COVID-19 exposure	.02	.01	.07	-.03	.02	.18	-.09	.02	<.001
Secondary stressors	.04	.02	.01	.02	.02	.17	.06	.02	<.001

Table S3. Standardized regression results for analyses across attitudes (N=5,661).

	Seriousness			Dismissive Attitudes			Response Efficacy		
Variables	*b*	*SE*	*p*	*b*	*SE*	*p*	*b*	*SE*	*p*
Journalistic Complexity	.07	.01	<.001	-.12	.01	<.001	.04	.01	.02
Ideological Focus	-.12	.01	<.001	.21	.01	<.001	-.15	.01	<.001
Domestic Focus	-.04	.01	<.001	.04	.02	.01	-.03	.01	.03
Non-news Information Channels	-.02	.01	.22	.01	.02	.70	-.04	.02	.03
Woman (0 = Man)	-.02	.01	.12	.01	.02	<.001	-.04	.02	.65
Race/Ethnicity (reference = White)									
Black/African American	-.02	.01	.22	-.05	.02	.78	.01	.02	.39
Other	.02	.02	.02	-.01	.02	<.001	.02	.02	.08
Hispanic	.03	.01	.09	-.06	.02	.78	.03	.02	.03
Age (reference = 18-29)									
30-44	.03	.02	.53	.01	.02	.47	.05	.02	.09
45-59	-.01	.02	.43	-.02	.02	.01	-.04	.02	.64
60+	.02	.02	<.001	-.07	.02	<.001	-.01	.03	<.001
Education (reference = No HS diploma)									
HS graduate or equivalent	.13	.02	.57	-.21	.03	.84	.09	.03	.37
Some college	.02	.04	.39	.01	.04	.29	-.04	.05	.67
BA and above	.03	.03	.02	.04	.03	.44	-.02	.04	.34
Region (reference = Northeast)									
Midwest	.08	.04	.01	-.03	.04	.20	.04	.05	.08
South	-.04	.02	.29	.03	.02	.25	-.04	.02	.16
West	.02	.02	.03	-.03	.02	.50	.04	.03	.27
Mental health diagnosis (0 = No diagnosis)	-.04	.02	.01	.01	.02	.01	-.03	.02	.05
Prior physical health ailments	.04	.01	.97	-.05	.02	<.001	.03	.02	.04
Media exposure	<.001	.01	.20	-.11	.02	.15	.04	.02	.92
Political party identification	.02	.02	<.001	-.02	.02	<.001	<.001	.02	<.001
Direct COVID-19 exposure	-.28	.02	.57	.32	.02	.13	-.25	.02	.23
Secondary stressors	.01	.01	.97	-.03	.02	.15	-.02	.02	.02

Table S4. Standardized regression results for analyses across domains of behavior (N=5,661).

	Health Behaviors			Risk Behaviors		
Variables	*b*	*SE*	*p*	*b*	*SE*	*p*
Journalistic Complexity	.09	.02	<.001	-.08	.02	<.001
Ideological Focus	-.14	.02	<.001	.10	.02	<.001
Domestic Focus	-.03	.02	.03	.03	.02	.07
Non-news Information Channels	-.06	.02	<.001	.03	.02	.15
Woman (0 = Man)	.06	.02	<.001	.03	.02	.90
Race/Ethnicity (reference = White)						
Black/African American	.01	.02	.69	<.001	.02	.01
Other	.04	.01	<.001	-.06	.02	<.001
Hispanic	.02	.02	.33	-.09	.02	.02
Age (reference = 18-29)						
30-44	-.01	.03	.62	-.06	.02	<.001
45-59	-.01	.03	.69	-.09	.03	<.001
60+	.03	.03	.27	-.09	.03	<.001
Education (reference = No HS diploma)						
HS graduate or equivalent	.04	.05	.45	-.16	.03	.92
Some college	.03	.05	.47	<.001	.05	.03
BA and above	.08	.05	.10	.10	.04	<.001
Region (reference = Northeast)						
Midwest	-.01	.02	.76	.14	.05	.01
South	.04	.03	.14	.06	.02	.69
West	.03	.02	.26	.01	.03	.11
Mental health diagnosis (0 = No diagnosis)	.03	.02	.06	-.04	.02	.01
Prior physical health ailments	<.001	.02	.94	-.05	.02	<.001
Media exposure	<.001	.02	.96	-.07	.02	.15
Political party identification	-.21	.02	<.001	.03	.02	<.001
Direct COVID-19 exposure	-.01	.01	.32	.17	.02	.11
Secondary stressors	.01	.02	.47	-.03	.02	<.001

Figure S3. Bivariate correlations between channels used among our national sample.

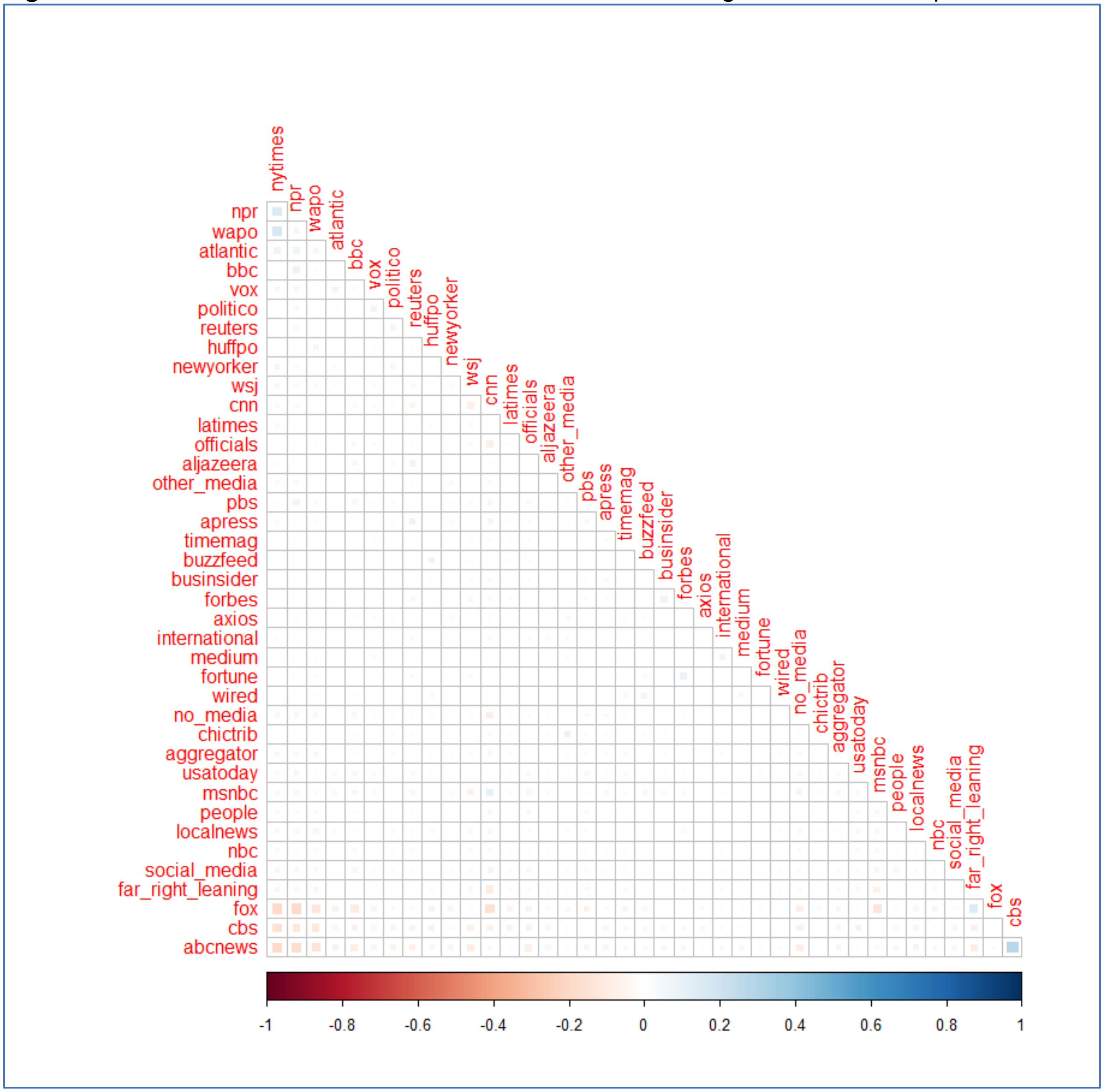

Supplement: Supplementary file 1 — Appendix 01 (PDF) [file pnas.2304550120.sapp.pdf]
